# Efficiency of a Multi-Soil-Layering System on Wastewater Treatment Using Environment-Friendly Filter Materials

**DOI:** 10.3390/ijerph120303362

**Published:** 2015-03-23

**Authors:** Chia-Chun Ho, Pei-Hao Wang

**Affiliations:** Department of Civil Engineering, National Taipei University of Technology, No. 1, Sec. 3, Chung-hsiao E. Rd., Taipei 10608, Taiwan

**Keywords:** multi-soil-layering system (MSL), zeolite, expanded clay aggregate, oyster shells, granular activated carbon

## Abstract

The multi-soil-layering (MSL) system primarily comprises two parts, specifically, the soil mixture layer (SML) and the permeable layer (PL). In Japan, zeolite is typically used as the permeable layer material. In the present study, zeolite was substituted with comparatively cheaper and more environmentally friendly materials, such as expanded clay aggregates, oyster shells, and already-used granular activated carbon collected from water purification plants. A series of indoor tests indicated that the suspended solid (SS) removal efficiency of granular activated carbon was between 76.2% and 94.6%; zeolite and expanded clay aggregates achieved similar efficiencies that were between 53.7% and 87.4%, and oyster shells presented the lowest efficiency that was between 29.8% and 61.8%. Further results show that the oyster shell system required an increase of wastewater retention time by 2 to 4 times that of the zeolite system to maintain similar chemical oxygen demand (COD) removal efficiency. Among the four MSL samples, the zeolite system and granular activated carbon system demonstrated a stable NH_3_-N removal performance at 92.3%–99.8%. The expanded clay aggregate system present lower removal performance because of its low adsorption capacity and excessively large pores, causing NO_3_^−^-N to be leached away under high hydraulic loading rate conditions. The total phosphorous (TP) removal efficiency of the MSL systems demonstrated no direct correlation with the permeable layer material. Therefore, all MSL samples achieved a TP efficiency of between 92.1% and 99.2%.

## 1. Introduction

Urbanisation increases the population density of residential areas, thus increasing the production of domestic wastewaters in these areas, which without proper treatment may severely affect the natural environment. Because of the ever-increasing awareness of environmental protection and sustainable management in recent years has prompted the Taiwanese government to aggressively seek cost-effective solutions for sewage treatment. In this context, the economical and easy-to-construct natural treatment system (NTS) has gained considerable attention in recent years. NTSs utilise natural elements, such as oxygen, soil, microbacteria, and plants, to purify wastewater to effluent standards before discharge into rivers and streams, thereby reducing water pollution caused by the discharge of domestic wastewaters. The most common NTS systems in Taiwan include constructed wetlands, cobble contact beds, and sand filters [1,2].

NTSs also present numerous limitations. For example, wastewater treatment using constructed wetlands is extremely time-consuming and requires a large area of land to achieve the desired treatment effects. Thus, this type of NTS becomes uneconomical in areas with high land costs. In addition, wetlands are typically areas with running water sources and are ideal habitats for mosquitoes and overgrown weeds and are therefore not favoured by residents. Regarding cobble contact beds and sand filters, fine solids often sediment within these systems, thus causing clogging. This reduces the treatment efficiency and increases maintenance difficulty. In 1990, Japan developed a novel wastewater treatment technique called the “multi-soil-layering system” (MSL). Compared with conventional soil treatment systems, this novel system could withstand a higher hydraulic loading rate (HLR) and was less prone to clogging [3]. The MSL system uses high quantities of natural, unpolluted materials to produce reusable water for eco-environment or agriculture [4]. Furthermore, this system can be maintained and operated at a low cost, requires only a small land area, and is ideal for urban areas in developing countries [5].

**Figure 1:**
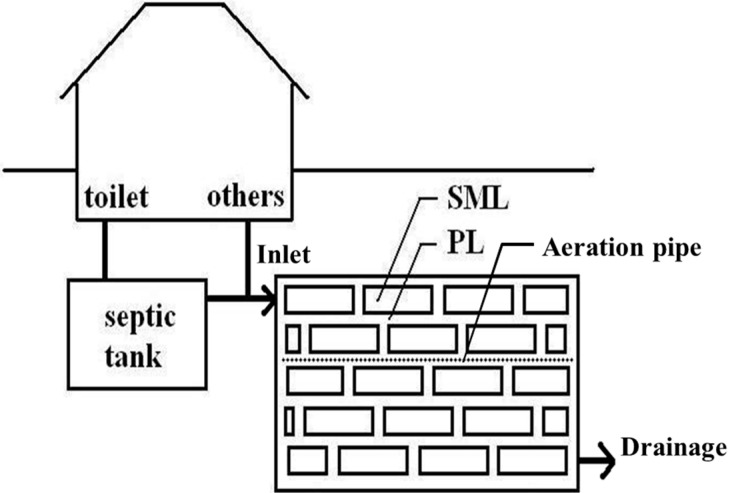
An MSL system for wastewater treatment of a single residential house.

The MSL system primarily comprises soil mixture layers (SMLs) and permeable layers (PLs). Figure 1 illustrates an MSL system created for wastewater treatment of a single residential house. The composition of the SML is approximately 70% to 80% soil and 20% to 30% additional materials, such as carbon powder, organic matter, and iron. Among the various materials that comprise the SML, the soil serves as a habitat for microorganisms; the carbon powder adsorbs high quantities of organic matter in wastewater, thus enhancing the efficiency of organic matter decomposition. The organic matter, such as sawdust, straw, corn cobs, and kenaf, serve as nutrients for microorganisms; furthermore, the iron materials effectively adsorb phosphates. The materials are mixed together and packed into fibre bags. The bags are then stacked to form the SMLs, with each layer separated by a PL. The PL comprises aggregates of gravel, pumice, or zeolite approximately 1–5 mm in diameter. Aggregates should be of consistent size to reduce the risk of clogging and facilitate the dispersion of water in the system. Moreover, the surface of the aggregates that constitute the PL also serves as habitats for nitrobacteria and adsorbs the organic matter in wastewater. Therefore, both layers actively remove pollutants from wastewater. In addition to the SMLs and PL, the MSL system is also equipped with inflow and outflow water pipes and a switchable perforated ventilation device, thus enabling the adjustment of the wastewater efficiency of the system by controlling aeration.

Establishing MSL systems is extremely economical because the constituents of the system, including soil, coal, sawdust, and metal, are locally available. Chen *et al.* [4] analyzed the cost of materials in China and pointed out that to construct an MSL system with a municipal wastewater treatment capacity of 100 m^3^/day at an HLR of 1 m^3^/m^2^/day, the required area is around 100 m^2^, with a depth of 1 m. If we assume that half of the system is composed of zeolite (100 m^3^), around 60 tons of zeolite would be required at a price of US$25/ton, for a total price of US$1500. The normal bulk density of SML is around 1.2 g/cm^3^ with sandy sand as the main material. The weight of SML is around 120 tons, out of which 70% is soil (around 84 tons). Charcoal, iron and sawdust comprise the other 30% of SML. The price for charcoal is around US$60/ton, sawdust US$25/ton and iron US$250/ton. Therefore, the whole cost for constructing such as MSL system can be less than US$10,000. Compared to conventional sewage systems and sewage treatment plants, the cost of operating and maintaining MSL systems is extremely low. Therefore, this type of NTS is an economical solution.

In an MSL system, the homogeneous coarse particles of PL enhance wastewater distribution and prevent clogging [6,7]. Zeolite has a high cation exchange capacity (CEC) and adsorption capacity; it also demonstrates highly active catalytic reactions as well as favourable acid resistance and thermal stability. Therefore, zeolite is commonly used in wastewater treatment [8,9,10,11]. Most Japanese studies related to MSL systems have thus adopted zeolite as the PL [12,13,14,15]. However, zeolite is not produced in Taiwan. Instead, Taiwan relies on imports for its acquisition, thus rendering the use of zeolite uneconomical and incompliant with the objective of using local materials. If a cheaper alternative that achieves the same water purification results as zeolite can be identified, then the overall cost for constructing MSL systems could be effectively reduced without compromising resource-recycling objectives.

## 2. Wastewater Treatment Mechanism of MSL

As wastewater passes through the MSL system, organic matter (examined using biochemical oxygen demand (BOD), chemical oxygen demand (COD), and organic nitrogen (Org-N) tests) adhere to the surface of the soil aggregates or PLs through physical or chemical mechanisms. The microorganism growing on these surfaces starts decomposing the organic matter, converting a portion of the Org-N in the wastewater into ammonium (NH_4_^+^-N). Regarding phosphate adhesion, the divalent ions from the iron materials in the SMLs dissolve under anaerobic conditions and are transferred to the surface of the SML and onto the PL. In an aerobic environment, the divalent ions oxidise into trivalent ions, and react with the phosphate ion in the wastewater, forming sediments. A high quantity of NH_4_^+^-N that enters the system is adsorbed by the SMLs and PL aggregates. Nitrification subsequently occurs in the aerobic environment, oxidizing the NH_4_^+^-N into nitrites (NO_2_-N) and nitrates (NO_3_-N) and releasing hydrogen ions that reduce the pH of the system. The NO_2_-N and NO_3_-N are then transported into the SMLs, in which denitrification occurs because of the anaerobic environment. The NO_2_-N and NO_3_-N are reduced to nitrogen (N_2_), nitrous oxide (N_2_O), and nitric oxide (NO). The reaction process consumes hydrogen ions, thus re-elevating the pH of the system. When this treatment mechanism is used, the pH value becomes an indicator for the ventilation conditions within the system. Appropriately adjusting the ventilation of the system facilitates decomposing NH_4_^+^-N and NO_3_-N, and eliminating BOD, COD, suspended solids (SS), and soluble reactive phosphorous (SRP) [4,16]. However, excessive ventilation suppresses denitrification, consequently reducing the removal efficiency of total nitrogen (TN) and total phosphorous (TP), and hindering the transfer of ferric hydroxide from the SMLs to the PLs [6,17]. Table 1 shows the removal characteristics of organic matter, phosphate, NH_4_^+^-N, and NO_3_-N in the MSL system.

**Table 1:** Removal characteristics of organic matter, phosphate, NH_4_^+^-N, and NO_3_-N in the MSL system.

Pollutant	Primary Reactant	Reaction Conditions	Precautions during Operation
Organic matter	Microorganism	Sufficient organic matter	The accumulated organic matter may cause microbial overgrowth, forming a biofilm that blocks water flow. The accumulation can be reduced by ventilating the system or leaving the system idle.
Phosphate	Fe(OH)_3_	Dissolution and oxidation of iron	During excessive ventilation, oxidation may cause Fe_2_O_3_ low-activation surfaces, which reduces effective surface area and phosphate fixation capacity.
NH_4_^+^-N NO_3_-N	Microorganism	Aerobic nitrification Anaerobic Denitrification	Aerobic and anaerobic states determine the removal of TN. These states can be controlled by adjusting the ventilation time and quantity.

Harada and Wakatsuki [18] developed an indoor livestock wastewater treatment model, the result of which demonstrated a favourable average waste removal rate at an HLR of 0.22 m^3^/m^2^/d, achieving a 96%–99% BOD removal, 95%–97% total suspended solid removal, 75%–99% TN removal, and 80%–99% TP removal. In addition, the findings of Masunaga *et al.* [19] indicated that under an HLR of between 0.03 and 0.29 m^3^/m^2^/d, the MSL system achieved removal rates of 600 g BOD/m^2^/d and 57.8 g N/m^2^/d for treating livestock wastewater, substantially higher than those of constructed wetlands under similar conditions. Attanandana *et al.* [16] reported that the filtration effects of natural wastewater treatment systems and subsurface flow wetlands are similar, both achieving removal rates of 2–30 g BOD/m^2^/d, 0.1–3 g N/m^2^/d, and 0.1–3 g P/m^2^/d. Similarly, MSL systems demonstrated removal rates of 113 g BOD/m^2^/d, 53 g N/m^2^/d, and 6.8 g P/m^2^/d, thus highlighting the efficiency of MSL systems in treating highly concentrated wastewater. Results also indicated that the MSL systems were 10 to 50 times more effective than were natural wastewater treatment systems and subsurface flow wetlands.

Most of the above mentioned studies used zeolite as PL for experiment. However, the amounts of zeolite used in these studies are high and costly. For this reason, zeolite was substituted with comparatively cheaper and more environmentally friendly materials, such as expanded clay aggregates, oyster shells, and already-used granular activated carbon collected from water purification plants, in an attempt to reduce the impact of waste on the environment, increase the use of renewable materials, and reduce the cost of establishing MSL systems. In the current study, the researchers implemented a self-developed test apparatus for examining the performance of the sample materials.

## 3. Materials and Method

### 3.1. Materials of the MSL System

The primary materials of the MSL system includes SMLs and PLs. For this study, the main purpose is to investigate the effectiveness of wastewater purification using four kinds of PL materials, zeolite, expanded clay aggregates, oyster shells, and already-used granular activated carbon; as a result, the same SMLs material is used for each experiment. The materials that constitute the MSL systems are listed as follows:

#### 3.1.1. Soil Mixture Layers, SMLs

Soil is the primary component of the SML. Other materials, such as powdered activated carbon, organic matter, and iron or aluminium, are added to the soil, and the mixture is packed into fibre bags. The fibre bags are then stacked in the treatment system with each layer separated by the PLs. The discussions of material properties used in the experiments are as follows.

##### *Soil* 

Soil influences phosphorous adsorption and microbial activity, with clay or loam demonstrating the most favourable effects. Wakatsuki *et al.* [6] indicated that the phosphorous adsorption potential of andisol soil was 1 g/kg, and that for quartz gravel was only 0.1 g/kg. For this reason, sandy loam soil with 0.121 mm of median particle size was used in this study.

##### *Powdered Activated* *Carbon*

Powdered activated carbon is a type of porous material with extremely large surface areas and particle sizes of less than 0.075 mm. Masunaga and Wakatsuki [17] suggested adding 10% powdered activated carbon into the SML. The activated carbon attracts organic matter as wastewater flows through the system, causing the organic matter to adhere to the carbon surface. This increases the processing efficiency of microorganisms, ultimately achieving waste removal.

##### *Organic* *Matter*

The main purpose of adding organic matter is to provide the carbon source for microorganisms. A comparative study on the effect of various organic components (sawdust, rice straw, kenaf, and corncob) on the efficiency of an MSL system for domestic wastewater treatment was reported, and the authors recommended adding 5% of organic matter into the SML [5]. In this study, Taiwanese common rice straw was used for experiments.

##### *Iron* *Scraps*

**Figure 2:**
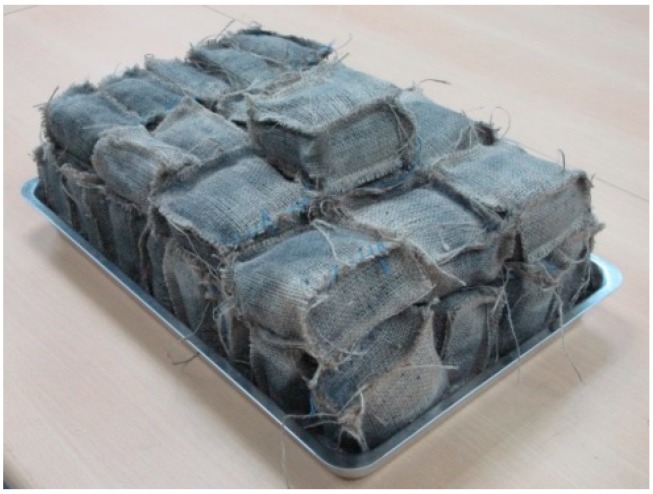
SML blocks.

Adding iron scraps into the SMLs facilitates phosphorus adsorption considerably. Wakatsuki *et al.* [6,20,21] reported that adding 10% iron scraps could increase phosphorus adsorption by 5–10 g/kg. The present study developed an MSL system by using materials commonly located in Taiwan in the aforementioned proportions. Based on the foregoing requirements, for the SML, sandy clay, powdered activated carbon, rice straws, and iron scraps were combined with a dry-weight ratio of 75%, 10%, 5%, and 10%, respectively. The mixture was then packed in gunnysacks (Figure 2).

#### 3.1.2. Permeable Layers, PLs

**Figure 3:**
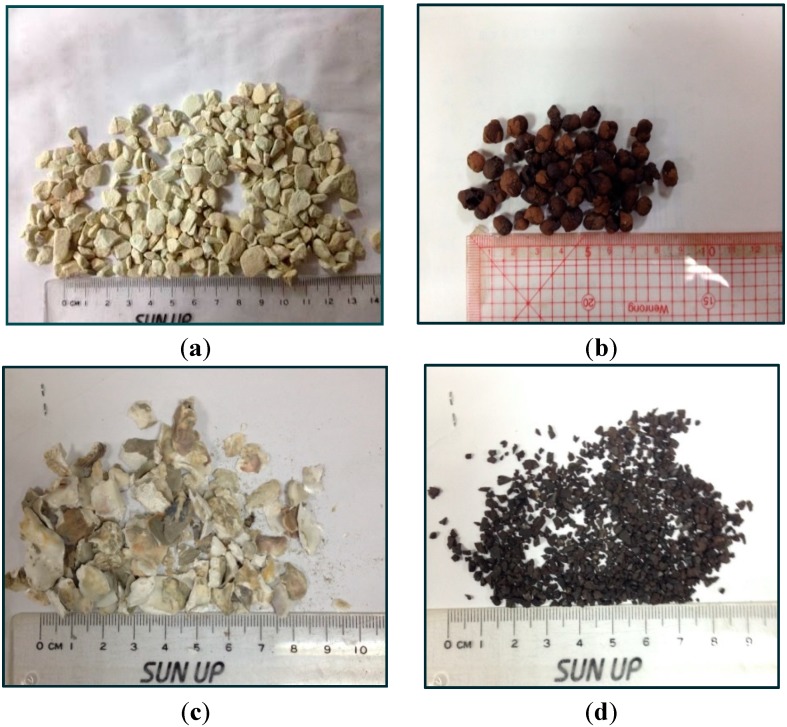
PL materials. (**a**) zeolite; (**b**) expanded clay aggregate; (**c**) oyster shells; (**d**) granular activated carbon.

The PL materials include gravel, pumice, perlite, or zeolite aggregate material [12,20,21]. In Japan, zeolite is typically used as the PL material. However, zeolite is considerably costly in Taiwan. In the present study, zeolite was substituted with comparatively cheaper and more environmentally friendly materials, such as expanded clay aggregates, oyster shells, and already-used granular activated carbon collected from water purification plants (Figure 3), in an attempt to reduce the impact of waste on the environment, increase the use of renewable materials, and reduce the cost of establishing MSL systems. The material properties of PLs are described below.

##### *Zeolite* 

Zeolites are aluminosilicate solids bearing a negatively charged honeycomb framework of micropores into which molecules may be adsorbed for environmental decontamination, and to catalyse chemical reactions [22]. Zeolite used in this study is a clinoptilolite type with a diameter ranging from 3–5 mm. Based on a CEC test, zeolite demonstrated favourable absorption ability (96 ± 8 meq/100 g) for the organic matter in wastewater.

##### *Expanded Clay* *Aggregate*

Expanded clay aggregate is also known as the lightweight aggregate and it is the sinter manufacture from sewage sludge ash or reservoir sludge. The expanded clay aggregates used in water purification is an innovative idea. The present study use the expanded clay aggregate from reservoir sludge with 4–6 mm of particle size and its CEC value is 47 ± 8 meq/100 g.

##### *Oyster* *Shells*

Oyster shell is a very common waste in Taiwan and has been used successfully in cobble contact bed. Therefore, this study attempts to apply it in the MSL system. The oyster shells were crushed and broken into particles of 5–6 mm and the CEC value is 32 ± 6 meq/100 g.

##### *Granular Activated* *Carbon*

Granular activated carbon has been utilized successfully and widely as filter medium of the water purification plants because of its high CEC and high adsorption capacity. However, the high cost is required to renew and restore the function of water purification of the already-used granular activated carbon. This study collected the already-used granular activated carbon from water purification plants and used in the MSL system. Its particle size is 2–3 mm. Four sample chambers were assembled using the identical SMLs with four PL materials, and a label was provided for each of the test samples; these labels are, System A (zeolite), System B (expanded clay aggregate), System C (oyster shells), and System D (granular activated carbon).

### 3.2. Test Apparatus

**Figure 4:**
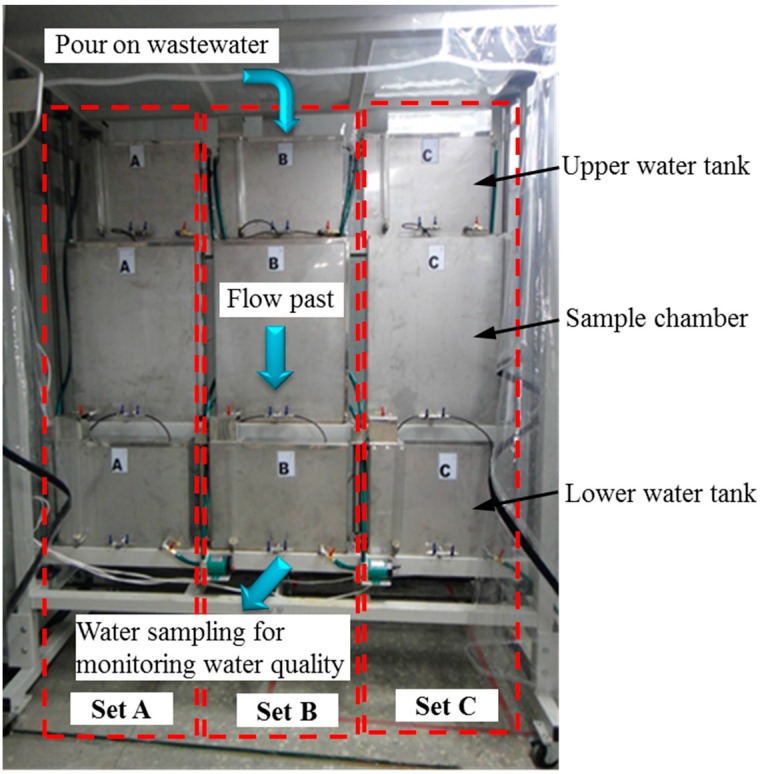
Indoor apparatus for testing the MSL systems.

A unique indoor test apparatus for examining various MSL systems was developed in this study. As shown in Figure 4, the apparatus consists of three sets of equipment A, B, and C. Each set of equipment comprised an upper water tank, lower water tank, and sample chambers. The upper water tank is a 500 mm (L) × 100 mm (W) × 500 mm (H) stainless steel structure primarily used for storing 0.025 m^3^ of wastewater. A bellow pipe with an adjustable nozzle is installed within the tank to control the inflow of wastewater into the sample chambers, thereby simulating the flow conditions in real-time scenarios. The middle section of the apparatus accommodates several sample chambers for the MSL systems. The wastewater enters these chambers from the upper water tank and into the MSL system. The chambers are closed stainless steel structures that are 500 mm (L) × 100 mm (W) × 700 mm (H) in size. The lower water tank is a 500 mm (L) × 100 mm (W) × 500 mm (H) stainless steel structure primarily used for collecting the wastewater after it passes through the MSL systems. The proposed apparatus can simultaneously test three MSL systems. Before testing, the pollution concentration of the wastewater was measured. Following testing, the pollution concentration of the water samples collected from the lower water tank was measured. The two sets of measurements were compared for determining the pollution removal efficiency of the MSL systems.

### 3.3. Experiment Process Scheme

In the current study, several tests were performed for examining the water purification efficiency of various PL materials. Therefore, the SML portion of the samples was identical. The surface water in the primary sedimentation tank at the Dihua Wastewater Treatment Plant in Taipei City was selected as the experimental wastewater. Because the wastewater was collected at different times, the wastewater concentration for each test differed. The test procedures are detailed in the following section.

#### 3.3.1. Preparing Test Samples (Sample Chamber)

**Figure 5:**
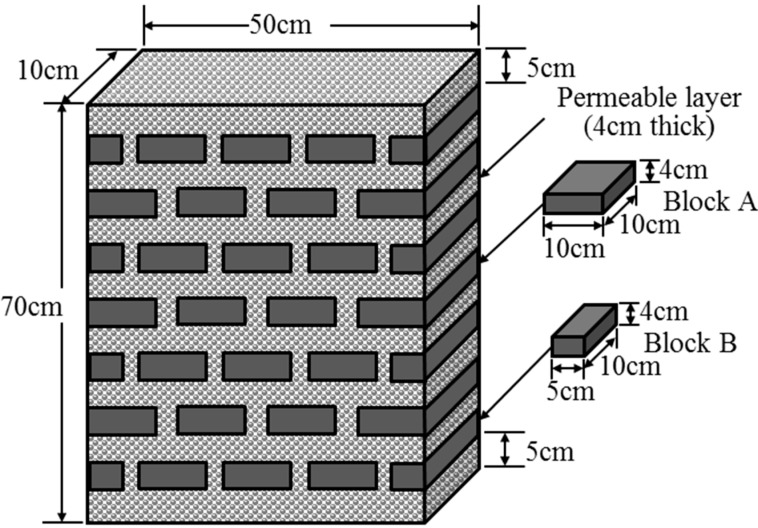
Material allocation within the sample chamber.

For stacking the SML blocks in an overlapping manner within the 50 cm (L) × 10 cm (W) × 70 cm (H) sample chamber, the SML blocks were prepared in two sizes. The blocks in the Block A group were 10 cm (L) × 10 cm (W) × 4 cm (H) in size, and those in the Block B group were 10 cm (L) × 5 cm (W) × 4 cm (H) in size. An SML followed by a PL was repeatedly stacked in the sample chamber to form the MSL system. First, a 5-cm-thick PL layer was paved at the base of the chamber followed by a 4-cm-thick SML layer, forming the base SML-PL layer. The horizontal intervals between the SML blocks were 2.5 cm, and this interval was filled with the PL material. Next, a 4-cm-thick PL followed by a 4-cm-thick SML were repeatedly packed onto the base layer. Finally, a 5-cm-thick PL was packed at the top of the chamber to complete assembling the sample chamber (Figure 5).

#### 3.3.2. Transferring Wastewater (Upper Water Tank)

The wastewater acquired from the wastewater treatment plant was transferred to the upper water tank. The inflow valve was opened, allowing the wastewater to enter the MSL system. Subsequently, the inflow velocity of the wastewater was controlled to observe the effects of the HLR on water purification. Four HLRs were observed, and these are 0.5, 1.0, 2.0, and 3.0 m^3^/m^2^/d.

#### 3.3.3. Monitoring Water Quality

The wastewater accumulated in the lower water tank after flowing through the sample chambers was extracted and tested for water quality. Test items include SS (APHA 20ed 2540D), COD (ASTM D1252-06), NH_3_-N (APHA 20ed 4500-NH_3_), and TP (APHA 21ed 4500-P).

## 4. Results and Discussion

Several tests were performed for each MSL sample under the same HLR conditions. Each test was performed following a four-to-seven-day interval and the test data were collected. The data were then analysed for determining the pollution removal efficiency of the samples.

### 4.1. SS Removal Efficiency

Table 2 shows the SS removal efficiency under various HLR conditions. The results indicated that Sample A achieved a 54.6% ± 5.0% SS removal when the HLR = 3.0 m^3^/m^2^/d; 65.4% ± 6.9% when the HLR = 2.0 m^3^/m^2^/d; 84.6% ± 8.9% when the HLR = 1.0 m^3^/m^2^/d; and 83.4% ± 10.8% when the HLR = 0.5 m^3^/m^2^/d. These results of four systems suggested that the SS removal rate increased as the HLR decreased. This is because SS are more effectively blocked by the MSL system when wastewater flows slowly through it. However, SS removal rates were similar under HLR = 1.0 and 0.5 m^3^/m^2^/d conditions. Thus, for System A, the optimal SS removal efficiency can be achieved when the HLR = 1.0 m^3^/m^2^/d.

**Table 2:** SS removal performance under different HLR conditions.

Test Condition		Filter Medium			
		System A	System B	System C	System D
HLR (m^3^/m^2^/d)	Inflow (mg/L)	11.28 ± 6.90	11.28 ±6.90	16.77 ± 5.65	16.77 ± 5.65
0.5	Outflow (mg/L)	1.80 ± 1.16	1.88 ± 1.31	6.24 ± 2.09	0.88 ± 0.26
	% removal	83.4 ± 10.8	82.5 ± 12.1	63.2 ± 4.7	94.5 ± 1.4
1.0	Outflow (mg/L)	1.57 ± 1.05	1.85 ± 1.19	7.56 ± 2.31	1.69 ± 0.75
	% removal	84.6 ± 8.9	82.8 ± 10.9	54.9 ±4.5	90.3 ± 2.9
2.0	Outflow (mg/L)	3.42 ± 1.80	4.16 ± 2.14	11.57 ± 4.62	2.12 ± 0.83
	% removal	65.4 ± 6.9	62.4 ± 11.4	33.5 ± 6.6	88.9 ± 1.8
3.0	Outflow (mg/L)	4.00 ± 1.86	4.38 ± 1.87	12.24 ± 4.67	3.97 ± 1.10
	% removal	54.6 ± 5.0	54.2 ± 11.5	32.4 ± 8.8	74.2 ± 4.9

**Figure 6:**
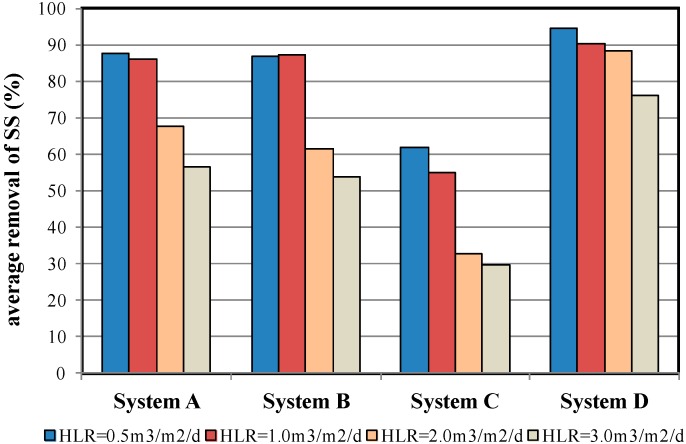
Average SS removal efficiency of the four MSL samples under various HLR conditions.

Figure 6 illustrates the average SS removal efficiency of the four MSL systems under various HLR conditions. The results indicated that System D achieved the most favourable SS removal efficiency, Systems A and B achieved similar efficiencies, and System C demonstrated the least favourable efficiency. The reason for the superior performance of System D was the small-sized granular activated carbon stacked in the system, which yielded the smallest pores compared with the other systems, thus increasing the filtration effect of SS. However, clogging is likely to occur in System D following extended use of the granular activated carbon. Zeolite and expanded clay aggregates were used in Systems A and B, respectively, and therefore produced similar blockage effects. Oyster shells were used in System C. This material presented the largest aggregate size among the four PL materials. The flat shape of oyster shells produced large, heterogeneous pores within the MSL system, which were unfavourable for blocking SSs. The test results clearly indicated that the SS removal efficiency was correlated to the aggregate size and shape of the PL material, and showed no direct correlation to the texture of the material. The test results also showed that the PL materials with an aggregate size of 3–6 mm achieved an SS removal efficiency exceeding 80% under HLR = 1.0 m^3^/m^2^/d conditions. Although the PL materials with smaller aggregate sizes can more effectively remove SS from wastewater, this study suggested that the HLR can be increased to reduce the chances of clogging within the MSL system.

### 4.2. COD Removal Efficiency

Table 3 shows the COD removal efficiency under various HLR conditions. The results indicated that the COD removal rate increased as the HLR decreased. This is because the retention time of the wastewater within the system increased as the HLR decreased, thus providing the system with sufficient time to adsorb, react, and remove the organic pollutants from the wastewater and consequently enhancing the removal efficiency [3]. In addition, the COD removal percentiles of System A under HLR = 0.5 and 1.0 m^3^/m^2^/d conditions were 76.9% ± 7.3% and 65.2% ± 11.9%, respectively, and those under HLR = 2.0 and 3.0 m^3^/m^2^/d conditions were 49.4% ± 14.7% and 30.5% ± 18.2%, respectively. This suggests that lower HLR conditions improved the stability of COD removal. System B to System D of experiments also showed the similar results.

**Figure 7:**
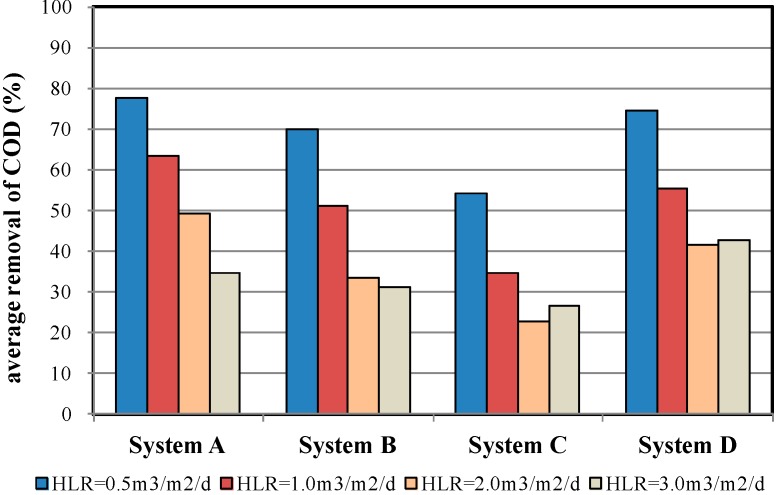
Average COD removal efficiency of the four MSL samples under various HLR conditions.

Figure 7 shows the average COD removal efficiency of the four MSL systems under various HLR conditions. The results showed that System A (Zeolite) achieved the most favourable efficiency, whereas System C demonstrated the least favourable efficiency. The researchers inferred that the superior performance of System A is attributed to the porous surface of the zeolite. Metal cations subsequently adhere within these pores and stimulate ion exchange. Zeolite demonstrated favourable absorption ability (96 ± 8 meq/100 g) for the organic matter in wastewater. Acclimated autotrophic bacteria on the zeolite then decompose the adsorbed organic matter to achieve COD removal. Granular activated carbon demonstrates similar effects with zeolite. However, the activated carbon used in the present study was the already-used activated carbon of a water purification plant. Because the activated carbon had been previously used, the pollutant adsorption ability of a portion of the aggregates had already reached saturation. Therefore, the newly employed zeolite material outperformed the activated carbon used in the present study. The oyster shell material used in System C demonstrated relatively lower CEC results (32 ± 6 meq/100 g), suggesting a weaker ion adsorption ability. Furthermore, oyster shells released salt into the wastewater as it passes through the system, thus increasing the salt content in the water. The pH values of the inflow wastewater were between 6.26 and 7.11 and those of the purified water (outflow) were between 6.69 and 7.57. This increase in pH is caused by the increased salt content, which inhibits microbial growth [23]. Therefore, the COD removal efficiency of System C was the lowest. A comparison of all four MSL samples showed that under HLR = 2.0 m^3^/m^2^/d conditions, System A achieved a COD removal rate of approximately 50%. Systems B, C, and D achieved similar results to System A (approximately 50%) at HLR = 1.0, 0.5–1.0, and 1.0–2.0 m^3^/m^2^/d, respectively. These findings suggest that the expanded clay aggregate, oyster shells, and granular activated carbon systems can achieve a similar COD removal efficiency as that of the zeolite system when the wastewater retention time within the MSL system is extended. Among these systems, the oyster shell system required a retention time two to four times that of the zeolite system.

**Table 3:** COD removal performance under different HLR conditions.

Test Condition		Filter Medium			
		System A	System B	System C	System D
HLR (m^3^/m^2^/d)	Inflow (mg/L)	170.7 ± 61.4	170.7 ± 61.4	203.2 ± 51.5	203.2 ± 51.5
0.5	Outflow (mg/L)	36.5 ± 9.1	51.1 ± 17.0	94.6 ± 25.1	52.8 ± 13.6
	% removal	76.9 ± 7.3	68.8 ± 7.5	54.6 ± 2.7	73.9 ± 4.1
1.0	Outflow (mg/L)	68.3 ± 29.1	78.3 ± 24.5	130.1 ± 32.8	85.4 ± 17.3
	% removal	65.2 ± 11.9	51.8 ± 4.1	33.9 ± 3.7	57.0 ± 2.7
2.0	Outflow (mg/L)	78.8 ± 15.6	110.6 ± 33.5	152.7 ± 35.4	111.6 ± 24.5
	% removal	49.4 ± 14.7	33.7 ± 4.2	22.9 ± 3.3	40.6 ± 6.0
3.0	Outflow (mg/L)	113.3 ± 41.0	114.8 ± 35.6	143.9 ± 29.8	119.5 ± 29.7
	% removal	30.5 ± 18.2	33.0 ± 8.6	26.8 ± 5.0	42.7 ± 3.1

Boonsook *et al.* [14] developed MSL systems and used zeolite, zeolitised perlite, perlite, gravel, and charcoal as the PL materials. They subsequently conducted a series of indoor tests and discovered that under an HLR of 0.096–0.346 m^3^/m^2^/d and under nonaerated conditions, all the PL materials achieved a COD removal rate of 79.0%–98.1%. For the present study, the average COD removal efficiency of the four MSL samples under HLR = 0.5 m^3^/m^2^/d is 77.8%, 69.8%, 54.1%, and 74.4%, respectively. It is obvious that the COD removal rate of System A and D are similar to the results of Boonsook *et al.*, but System B and C show a poor performance. Therefore, it is required to reduce HLR to improve the performance of System B and C.

### 4.3. NH_3_-N Removal Efficiency

The removal of N relies on microbial actions. Specifically, Org-N is converted into NH_3_-N through ammonification. Next, NH_3_-N is converted into NO_2_-N, and subsequently into NO_3_-N, through nitrification. Finally, NO_2_-N and NO_3_-N are converted into N_2_ through denitrification [4]. Table 4 shows the NH_3_-N removal efficiency under various HLR conditions. Zeolite demonstrated favourable adsorption and ion exchange abilities for NH_4_^+^-N. In addition, zeolite showed a higher porosity, larger surface area, and coarser aggregate surface than those of the other sample materials, and is therefore a more favourable carrier for microorganisms. The zeolite material in the MSL system adsorbs the NH_4_^+^-N ions in the wastewater through ion exchange mechanism and serves as a carrier for nitrifying bacteria, which convert the NH_3_-N in the wastewater into NO_3_-N, thereby forming a self-adsorbing/self-nitrifying circulation process. In other words, the zeolite material achieves continuous circulation through a full or partial self-regeneration process. Therefore, System A achieved an NH_3_-N removal efficiency that ranged from 87.4% to 99.8% under various HLR conditions. Performance on NH_3_-N removal of System B and System D were significantly worse than System A.

**Table 4:** NH_3_-N removal performance under different HLR conditions.

Test Condition		Filter Medium			
		System A	System B	System C	System D
HLR (m^3^/m^2^/d)	Inflow (mg/L)	24.6 ± 3.7	24.6 ± 3.7	27.5 ± 6.9	27.5 ± 6.9
0.5	Outflow (mg/L)	0.07 ± 0.05	2.82 ± 0.79	6.26 ± 1.59	0.27 ± 0.14
	% removal	99.7 ± 0.2	87.2 ± 4.5	77.9 ± 1.6	99.2 ± 0.4
1.0	Outflow (mg/L)	0.08 ± 0.06	7.73 ± 0.89	12.91 ± 2.62	0.22 ± 0.12
	% removal	99.7 ± 0.2	67.0 ± 5.2	53.8 ± 3.9	99.3 ± 0.4
2.0	Outflow (mg/L)	0.37 ± 0.19	14.15 ± 2.37	16.54 ± 4.44	0.57 ± 0.18
	% removal	98.6 ± 0.7	45.6 ± 4.0	41.3 ± 1.9	97.6 ± 1.3
3.0	Outflow (mg/L)	2.96 ± 1.00	14.63 ± 2.74	20.19 ± 4.82	1.72 ± 0.41
	% removal	87.5 ± 4.5	37.2 ± 5.9	24.8 ± 2.5	93.4 ± 2.0

**Figure 8:**
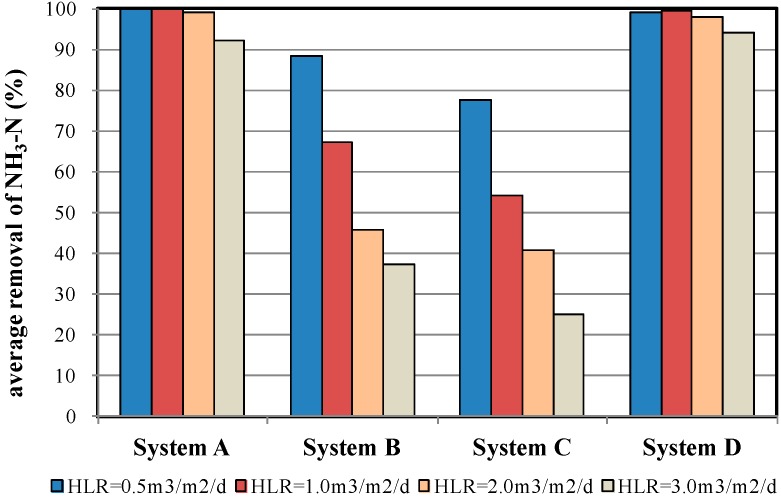
Average NH_3_-N removal efficiency of the four MSL samples under various HLR conditions.

In addition to System A, System D also demonstrated a favourable NH_3_-N removal efficiency (Figure 8). The characteristics of the granular activated carbon used in System D were similar to those of zeolite; specifically, the aggregate surface of the activated carbon demonstrated favourable ion adsorption ability and was rich with microorganisms. Therefore, the activated carbon could simultaneously achieve chemisorption, ion exchange, and bionitrification. However, the activated carbon used in the present study was recycled from a water purification plant and thus the pollutant adsorption ability of a portion of the aggregates had already reached saturation, thus resulting in a slightly weaker NH_3_-N removal efficiency than that of System A. However, System D still achieved an NH_3_-N removal efficiency of more than 90% under various HLR conditions.

**Figure 9:**
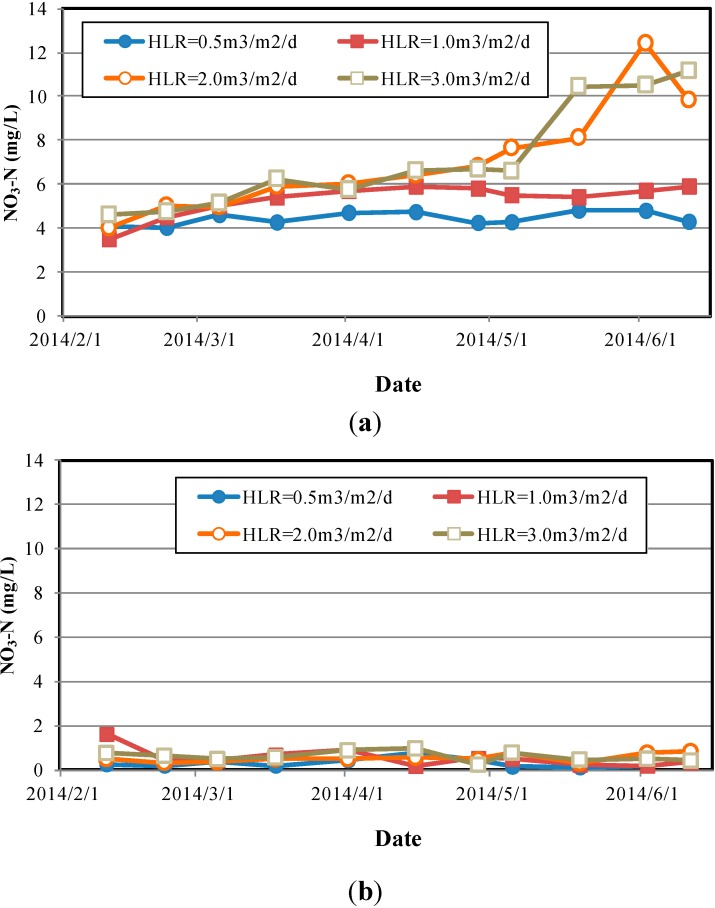
Changes in outflow NH3-N concentrations under various HLR conditions. (**a**) System B (expanded clay aggregate); (**b**) System A (zeolite).

Systems B and C demonstrated significantly lower NH_3_-N removal efficiencies under all HLR conditions, except for HLR = 0.5 m^3^/m^2^/d, under which the systems maintained a 70% to 80% removal efficiency. Performance was particularly low under high HLR conditions, at which the systems demonstrated removal efficiencies of 50% or less. The primary reason for the lack of efficiency was the lower CEC values of the expanded clay aggregates (47 ± 8 meq/100 g) and oyster shells (32 ± 6 meq/100 g). Moreover, expanded clay aggregates are macroporous, lightweight aggregates with a relatively weaker ion adsorption ability. Under high-flow velocity conditions, the NO_3_^−^-N attached to the surface and within the pores of the aggregates are easily leached away, thus increasing the NO_3_^−^-N concentration in the outflow. Therefore, the NO_3_^−^-N concentrations in the outflow samples increased over time. These results were consistent with those obtained by Boonsook *et al.* [14], who tested a zeolitised perlite-based MSL system. As shown in Figure 9a, the increase in NO_3_^−^-N concentrations over time was less apparent under low HLR conditions. As illustrated in Figure 9b, the outflow NO_3_^−^-N concentrations of System C were significantly higher than those of System A, indicating that the denitrification of the zeolite system outperformed that of the expanded clay aggregate system.

### 4.4. TP Removal Efficiency

**Figure 10:**
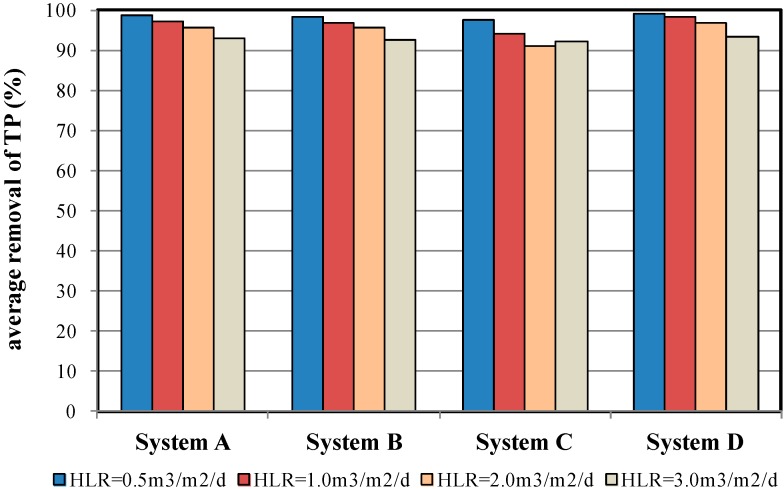
Average TP removal efficiency of the four MSL samples under various HLR conditions.

The removal of TP primarily relies on the iron scraps and soil within the SML blocks. Phosphate ions combine with the iron ions in the SML blocks, causing the sedimentation of the phosphate ions. This removal mechanism demonstrated no direct correlation with the selected PL material [4]. As shown in Figure 10, all four MSL samples achieved a TP removal efficiency higher than 90%. For example, System A achieved TP removal efficiencies of 98.7%, 97.1%, 95.8%, and 93.1% under HLR = 0.5, 1.0, 2.0, and 3.0 m^3^/m^2^/d conditions, respectively. The results also indicated that the TP removal efficiency decreased slightly as the HLR conditions increased. Therefore, when the HLR is controlled, and when an appropriate amount of iron scraps is added to the SML, MSL systems can effectively remove TP from wastewater regardless of the PL material selected. The TP removal efficiency under various HLR conditions was shown Table 5. The results also indicated that the TP removal rate increased as the HLR decreased.

**Table 5:** TP removal performance under different HLR conditions.

Test Condition		Filter Medium			
		System A	System B	System C	System D
HLR (m^3^/m^2^/d)	Inflow (mg/L)	7.9 ± 3.8	7.9 ± 3.8	10.7 ± 5.0	10.7 ± 5.0
0.5	Outflow (mg/L)	0.15 ± 0.12	0.14 ± 0.06	0.28 ± 0.19	0.08 ± 0.04
	% removal	98.5 ± 0.8	98.4 ± 0.4	97.8 ± 0.7	99.1 ± 0.1
1.0	Outflow (mg/L)	0.25 ± 0.12	0.27 ± 0.16	0.57 ± 0.36	0.12 ± 0.03
	% removal	96.9 ± 1.2	96.4 ± 0.5	94.4 ± 2.3	98.3 ± 0.6
2.0	Outflow (mg/L)	0.35 ± 0.16	0.37 ± 0.14	0.90 ± 0.37	0.49 ± 0.35
	% removal	95.8 ± 1.7	95.6 ± 0.6	90.5 ± 1.4	96.3 ± 1.5
3.0	Outflow (mg/L)	0.45 ± 0.24	0.63 ± 0.30	0.80 ± 0.31	0.58 ± 0.29
	% removal	92.5 ± 3.1	93.2 ± 1.2	91.4 ± 2.3	93.3 ± 1.6

## 5. Conclusions

The present study developed an MSL test apparatus and conducted a series of indoor tests, determining that the PL material influenced the water purification efficiency. Zeolite and granular activated carbon are porous materials with consistent aggregate sizes, coarse surfaces, and favourable CEC values, making them ideal for removing SS, COD, and NH_3_-N. Therefore, this study maintained that substituting zeolite with recycled granular activated carbon as the PL material in MSL systems is feasible. However, extended use of granular activated carbon may cause clogging because of its relatively smaller aggregate size. To prevent clogging, this study suggests increasing the HLR. Moreover, expanded clay aggregates are macroporous, lightweight aggregates with relatively weaker ion adsorption ability. For improving the COD and NH_3_-N removal efficiency of expanded clay aggregates, using an HLR of between 0.5 and 1.0 m^3^/m^2^/d is recommended. Oyster shells are large, flat aggregates that form unevenly distributed pores within the MSL system, which are unfavourable for the blockage of SS. The low CEC of oyster shells also indicates that this material possesses unfavourable ion adsorption ability. In addition, oyster shells release salt into the wastewater as it flows through the system. The increased salt content consequently suppresses microbial growth. Thus, oyster shells demonstrated the lowest average COD and NH_3_-N removal efficiencies among the four MSL samples. Overall, pollutant removal can still be achieved using oyster shells as the PL material, but the retention time of the wastewater within the MSL system should be lengthened. This study suggests using an HLR < 0.5 m^3^/m^2^/d. Moreover, all four PL materials achieved favourable TP removal efficiencies. To sum up, zeolite can be successfully substituted with expanded clay aggregates, oyster shells, and already-used granular activated carbon collected from water purification plants under the lower HLR and can reduce the impact of waste on the environment, increase the use of renewable materials, and reduce the cost of establishing MSL systems.

## References

[1] Chen C.F., Lin J.Y., Huang C.H., Chen W.L., Chueh N.L. (2009). Performance evaluation of a full-scale natural treatment system for nonpoint source and point source pollution removal. Environ. Monit. Assess..

[2] Kao C.M., Wang J.Y., Lee H.Y., Wen C.K. (2001). Application of a constructed wetland for non-point source pollution control. Water Sci. Technol..

[3] Masunaga T., Sato K., Shirahama M., Kudo H., Wakatsuki T. (2007). Characteristics of wastewater treatment using a multi-soil-layering system in relation to wastewater contamination levels and hydraulic loading rates. Soil Sci. Plant Nutr..

[4] Chen X., Luo A.C., Sato K., Wakasuki T., Masunaga T. (2009). An introduction of a multi-soil-layering system: A novel green technology for wastewater treatment in rural areas. Water Environ. J..

[5] Luanmanee S., Boonsook P., Attanandana T., Saitthiti B., Panichajakul C., Wakatsuki T. (2002). Effect of intermittent aeration regulation of a multi-soil-layering system on domestic wastewater treatment in Thailand. Ecol. Eng..

[6] Wakatsuki T., Esumi H., Omura S. (1993). High performance and N&P removable on-site domestic waste water treatment system by Multi-Soil-Layering method. Water Sci. Technol..

[7] Wakatsuki T., Luanmanee S., Masunaga T., Attanandana T. High rade on-site treatment of domestic wastewater and polluted river water by Multi-Soil-Layering method. Proceedings of the IWA (International Water Association) Conference.

[8] Wen D., Ho Y.S., Tang X. (2006). Comparative sorption kinetic studies of ammonium onto zeolite. J. Hazard. Mater..

[9] Saltali K., Sari A., Aydin M. (2007). Removal of ammonium ion from aqueous solution by natural Turkish (Yildizeli) zeolite for environmental quality. J. Hazard. Mater..

[10] Karapinar N. (2007). Application of natural zeolite for phosphorus and ammonium removal from aqueous solutions. J. Hazard. Mater..

[11] Cintoli R., Sabatino B.D., Galeotti L., Bruno G. (1995). Ammonium uptake by zeolite and treatment in UASB reactor of piggery wastewater. Water Sci. Technol..

[12] Sato K., Masunaga T., Wakatsuki T. (2005). Water movement characteristics in a Multi-Soil-Layering system. Soil Sci. Plant Nutr..

[13] Sato K., Masunaga T., Wakatsuki T. (2005). Characterization of treatment processes and mechanisms of COD, phosphorus and nitrogen removal in a multi-soil-layering system. Soil Sci. Plant Nutr..

[14] Boonsook P., Luanmanee S., Attanandana T., Kamidouzono A., Masunaga T., Wakatsuki T. (2003). A comparative study of permeable layer materials and aeration regime on efficiency of multi-soil-layering system for domestic wastewater treatment in Thailand. Soil Sci. Plant Nutr..

[15] Pattnaik R., Yost R.S., Porter G., Masunaga T., Attanandana T. (2007). Improving Multi-Soil-Layering (MSL) system remediation of dairy effluent. Ecol. Eng..

[16] Attanandana T., Saitthiti B., Thongpae S., Kritapirom S., Luanmanee S., Wakatsuki T. (2000). Multi-media-layering system for food service wastewater treatment. Ecol. Eng..

[17] Masunaga T., Wakatsuki T. High quality water remediation by the Multi Soil Layering Method. Proceedings of the 12th International Conference on Chemistry for Protection of the Environment.

[18] Harada K., Wakatsuki T. Advanced treatment for livestock wastewater by MSL system. Proceedings of the 31st Annual Conference of Japanese Society of Water Environment Tokyo, Japanese Society of Water Environment.

[19] Masunaga T., Sato K., Wakatsuki T. Environmental remediation using purification function of soil by Multi-Soil-Layering system. Proceedings of the 17th World Congress of Soil Science, Symposium 55.

[20] Wakatsuki T., Omura S., Abe Y., Izumi K. (1989). Treatment of domestic waste water by Multi-Soil-Layering method. Jpn. J. Soil Sci. Plant. Nutr..

[21] Wakatsuki T., Omura S., Abe Y., Izumi K. (1990). Role and life of purification abilities of soil materials in the MSL system. Jpn. J. Soil Sci. Plant. Nutr..

[22] Rhodes C.J. (2010). Properties and applications of zeolites. Sci. Prog..

[23] Rinzema A., van Lier J., Lettinga G. (1988). Sodium inhibition of acetoclastic methanogens in granular sludge from a UASB reactor. Enzyme Microb. Technol..

